# Constructing Carbon Fiber Motion-Detection Loops for Simultaneous EEG–fMRI

**DOI:** 10.3389/fneur.2014.00260

**Published:** 2015-01-05

**Authors:** David F. Abbott, Richard A. J. Masterton, John S. Archer, Steven W. Fleming, Aaron E. L. Warren, Graeme D. Jackson

**Affiliations:** ^1^The Florey Institute of Neuroscience and Mental Health, Austin Hospital, Melbourne, VIC, Australia; ^2^The University of Melbourne, Melbourne, VIC, Australia; ^3^Austin Hospital, Melbourne, VIC, Australia

**Keywords:** EEG–fMRI, motion detection, cardioballistic artifact, cardioballistic artefact, gradient artifact, gradient artefact, artifact removal, artefact removal

## Abstract

One of the most significant impediments to high-quality EEG recorded in an MRI scanner is subject motion. Availability of motion artifact sensors can substantially improve the quality of the recorded EEG. In the study of epilepsy, it can also dramatically increase the confidence that one has in discriminating true epileptiform activity from artifact. This is due both to the reduction in artifact and the ability to visually inspect the motion sensor signals when reading the EEG, revealing whether or not head motion is present. We have previously described the use of carbon fiber loops for detecting and correcting artifact in EEG acquired simultaneously with MRI. The loops, attached to the subject’s head, are electrically insulated from the scalp. They provide a simple and direct measure of specific artifact that is contaminating the EEG, including both subject motion and residual artifact arising from magnetic field gradients applied during MRI. Our previous implementation was used together with a custom-built EEG–fMRI system that differs substantially from current commercially available EEG–fMRI systems. The present technical note extends this work, describing in more detail how to construct the carbon fiber motion-detection loops, and how to interface them with a commercially available simultaneous EEG–fMRI system. We hope that the information provided may help those wishing to utilize a motion-detection/correction solution to improve the quality of EEG recorded within an MRI scanner.

## Safety Warning

This technical note describes construction and application of carbon fiber motion-detection leads. We have used these for simultaneous EEG–fMRI experiments, where a number of safety measures that are not detailed in this document have been taken to avoid inducing large currents causing injury. If you are considering use of similar equipment in an environment such as an MRI scanner then it is essential that you understand the safety implications. We recommend you to consult the literature for further information, for example, Ref. (1–3). We have used our leads with an EEG system that we developed in-house, and with commercially available systems. However, there is no guarantee that these leads will work properly with your EEG equipment. If you wish to use similar leads with a commercial EEG system then you should consult the manufacturer to ensure that there are no additional compatibility or safety issues. You should conduct your own testing to ensure the safety of the leads in your desired application.

## Disclaimer

The authors do not warrant the quality, accuracy, completeness, or suitability of any information in this note. The information is provided “as is” without representations, warranties, or conditions of any kind, express, or implied. Your use of any information herein is entirely at your own risk. In no event shall we be liable for any damages whatsoever, including special, indirect, or consequential damages, arising out of or in connection with the use of information in this note.

## Introduction

This technical note is provided to assist those wishing to construct carbon fiber motion-detection loops for use with simultaneous EEG–fMRI apparatus. Please read and understand both the safety warning and disclaimer above. Wires in an MRI scanner can be very dangerous if the proper precautions are not taken; these precautions are beyond the scope of this note.

Motion (including cardioballistic) artifact can be measured using insulated carbon fiber loops that are physically but not electrically attached to the subject’s head. The signals generated by small movement of these wires in the magnetic field are then used to estimate and remove motion artifact from the EEG. We have already described, demonstrated, and validated the approach, elsewhere (4). The purpose of the current note is to assist those wishing to build their own motion detector loops as, at the time of writing, we are not aware of an equivalent commercially available product. We also describe how these motion loops can be used in conjunction with commercially available MRI-compatible EEG equipment. Adaption to a commercial system incorporates additional processing steps to minimize the impact of motion on gradient artifact reduction. These steps were not required for our custom EEG system as it avoids the gradient artifact during the EEG recording. In currently available commercial systems, the gradient artifact is fully recorded by the EEG system and the subsequent average-artifact correction techniques can be confounded by subject motion artifact.

Availability of direct motion artifact sensors can substantially improve the quality of the recorded EEG. In the application to which we most often use the system – the study of epilepsy, it can also dramatically increase the confidence that we have in discriminating true epileptiform activity from artifact. This is due both to the reduction in artifact and the ability to visually inspect the motion sensor signals when reading the EEG, revealing whether or not motion is present (5). Aside from motion-detection loops, other methods for microscopic subject motion detection may be suitable to reduce motion artifact in EEG acquired in an MRI scanner. For example, a promising optical moiré phase tracking method has recently been proposed (6). However, the carbon fiber loops that we describe herein confer the advantage of being a simple direct measure of specific artifact that is contaminating the EEG, including both subject motion and residual artifact arising from magnetic field gradients applied during MRI.

Carbon fiber electrodes have been constructed at our institute and used in-house with our 3 T MRI scanner since the year 2000 with no adverse effects; for example, Ref. (7–14). We have also used carbon fiber cables and electrodes for intracranial EEG in sheep (15). Subsequently, we developed motion detector loops (4), which are carbon fiber loops constructed in a similar manner to our electrode leads. We began using these loops in conjunction with our own in-house-built MRI-compatible EEG recording equipment, for example, Ref. (4, 5, 16–24) and more recently we adapted them for use with a commercially available MRI-compatible EEG recorder (BrainAmps, BrainProducts, Germany); it is these particular leads that we describe in this note.

## Methods (Loop Construction)

The lead is constructed from bundles of carbon fiber thread (~1 mm bundles) enclosed in 2 mm diameter polyethylene tubing. At the amplifier end, the carbon fiber is crimped onto an appropriate connector to interface with the amplifier (e.g., a standard 1.5 mm touch-proof medical connector).

### Key components

#### Carbon fiber

**Figure 1:**
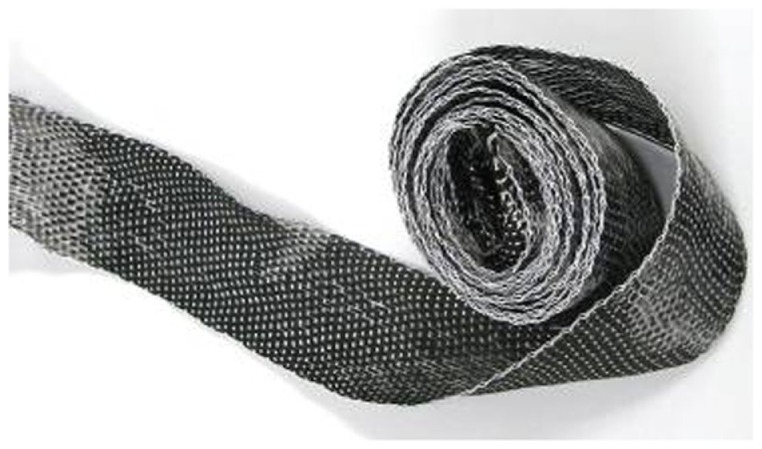
**Roll of carbon fiber tape from which bundles of “wire” can be extracted**.

This comes in many forms, but to make our “wire” we use weaved mats or tape, and small (1–2 mm diameter) bundles can be extracted from these. The length of the bundle used to make the wire will be determined by their intended use. We routinely constructed 2 m lengths of wire to reach from the back of our MRI scanner to the patient’s head. We have managed to source long enough offcuts on eBay as needed (Figure 1).

#### Polyethylene tubing

The carbon fiber will be threaded into lengths of PE tubing. We use 2 mm diameter tubing that we obtained from Microtube Extrusions Pty Ltd. in Australia [PE tubing 2.08 mm × 1.57 mm, rolls (30 m), Product Code PE208157]^1^.

#### Ferrules

**Figure 2:**
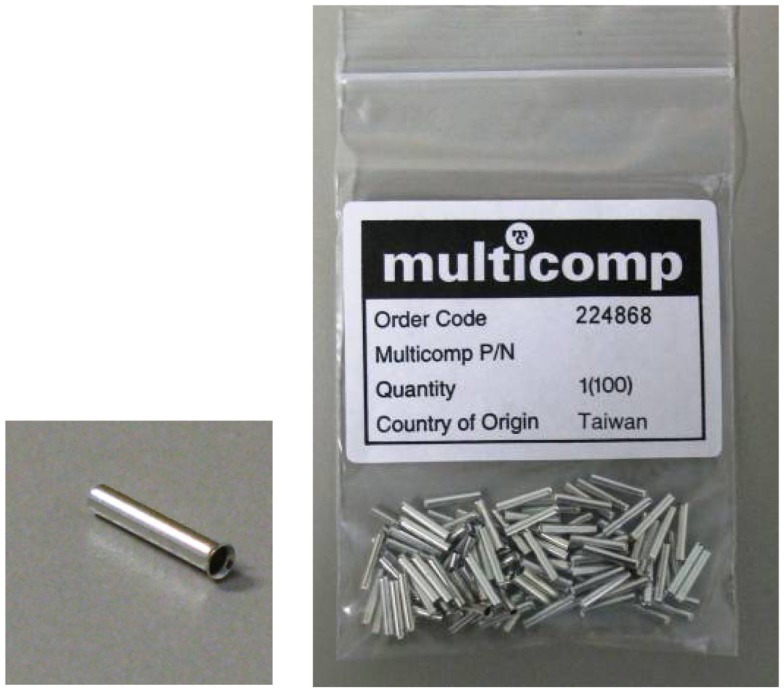
**Ferrules**.

In order to make an electrical and physically robust connection between the carbon fiber and any metal components such as resistors, RF absorbers, or wire, we use ferrules (Figure 2) that can be fitted into the PE tubing and crimped down onto the carbon fiber and metal component. We obtain ferrules from element14 Pty. Ltd. in Australia [Ferrule, 1.0 mm (Packs of 100) Order Code: 224868]^2^.

#### RF absorber

In order to reduce radiofrequency contamination of the signal, we attach an RF absorber to the electrode at the amplifier end of each insulated carbon fiber wire (this is shown later in Figure 10F). We use a Chomerics CHO-DROP^®^ EMI absorber (part number 80-10-9714-1000), which has a specified insertion loss of 15 dB at 100 and 150 MHz. We obtain these from element14 Pty. Ltd. in Australia (Order Code: 152658)^2^.

#### Resistors

We place a resistor of 32 kΩ in series with each motion loop. The value was chosen conservatively to be at least twice the measured DC resistance of a circuit consisting of two of the EEG electrodes supplied with the BrainAmps system. This resistor dominates the circuit, as the carbon fiber lead resistance is of the order of just a couple of 100 Ω.

#### Connectors

**Figure 3:**
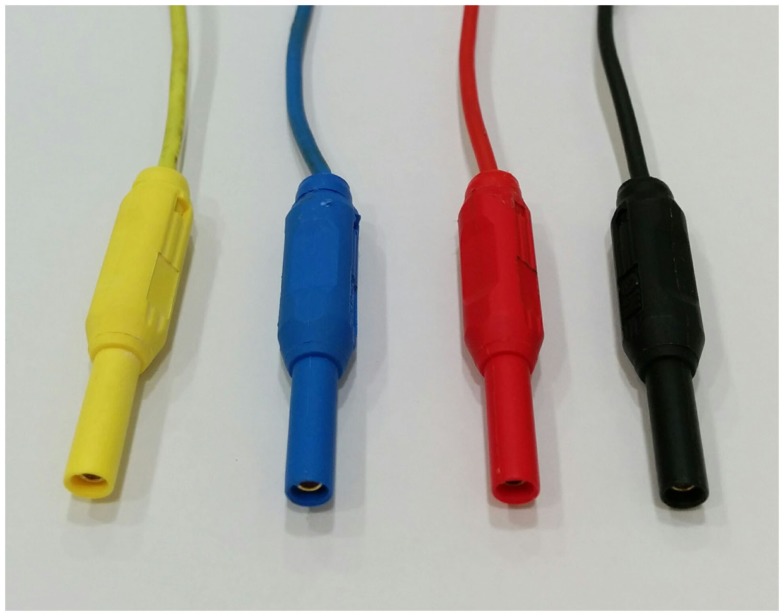
**Touch-proof safety connectors**.

To connect the insulated carbon fiber wires to the amplifier, we use individual standard touch-proof safety connectors (Figure 3) sourced from element14 Pty. Ltd. in Australia [Touch-proof Plugs (typical EEG safety connectors); Pack Type: Black, Red (Packs of 4) Order Code: 41300, Pack Type: Multicolor (Packs of 6) Order Code: 1085511].

### Construction

We first determine the length desired for the cables. We require different length wires for our different scanners; this depends on the room and scanner configuration. We then cut the carbon fiber tape and PE tubing to the appropriate length. We allow a little extra at this stage to allow for braiding of the cables later.

#### Making the cable

**Figure 4:**
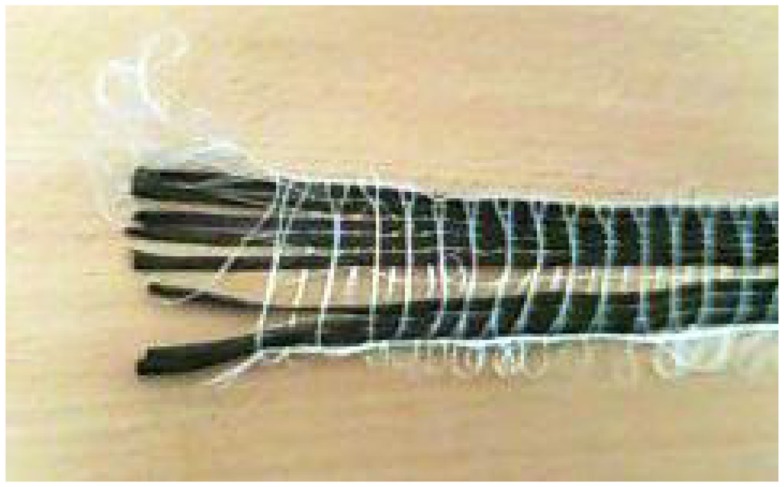
**Extracting carbon fiber bundles from woven tape**.

**Figure 5:**
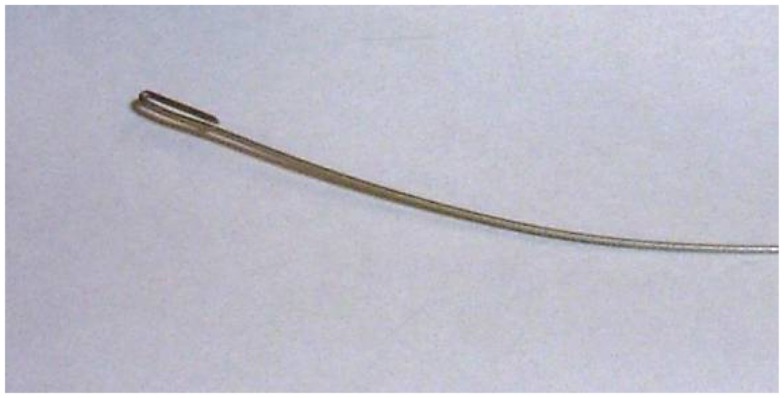
**Guide wire used to help thread carbon fiber through the PE tubing**.

Carbon fiber tape often comes in a simple weave, and it is possible to extract the fiber in bundles, a millimeter or so in diameter (Figure 4). Inserting the fiber into the PE tubing is one of the more tedious parts of the job. We create a simple guide wire to help thread the carbon fiber (Figure 5). The guide wire is a long piece of thin, fairly stiff wire that can be threaded through the PE tubing. The wire must be longer than the PE tubing and much thinner than the diameter of the bore of the PE tubing. We bend a tight hook onto one end of the wire in order to catch the carbon fibers and drag them back through the PE tubing.

Since we need to pull the carbon fiber and wire back through the tubing, it works best if the wire is quite thin and has no kinks in it. Any kinks will increase the friction, and make the job a lot harder, and it may even result in perforation or tearing of the PE tubing. It is very important that the tubing has no holes in it where current may leak as that would be a significant patient safety problem.

**Figure 6:**
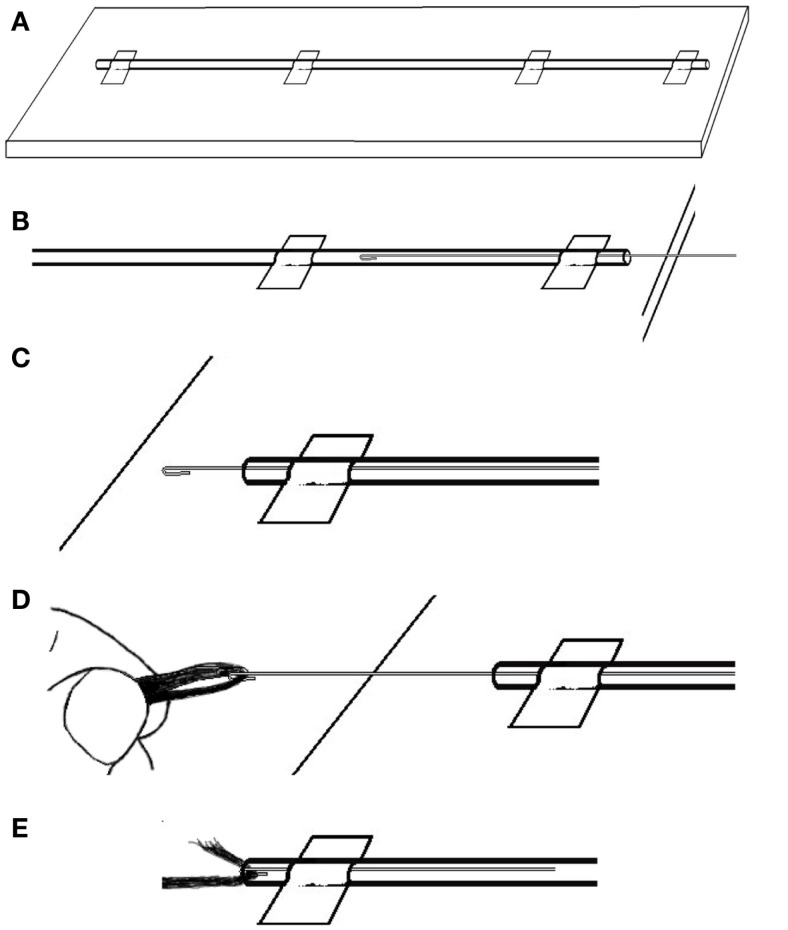
**To thread the guide wire through the PE tubing: (A) tape the tubing in a straight line then (B) carefully push the wire through the PE tubing until (C) it comes out the other end**. Then **(D)** catch the end of the length of carbon fiber with the wire hook and **(E)** carefully pull the fiber into and then all the way through the PE tubing.

**Figure 7:**
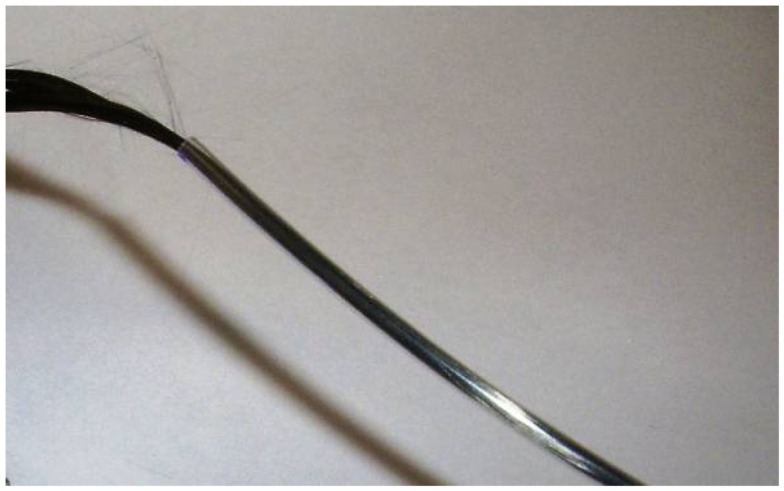
**Carbon fiber successfully threaded through PE tubing**.

We find the easiest way to thread the carbon fiber is to first lay out the length of PE tubing on a long table. It will work best if the table is longer than the intended cable. We tape the tubing in a straight line (Figure 6A), then carefully push the wire through the PE tubing until it comes out the other end (Figures 6B,C). Then, we catch the end of the length of carbon fiber with the wire hook and carefully pull the fiber all the way through the PE tubing (Figures 6D,E). Once we have an insulated carbon fiber cable (Figure 7), we visually check the cable closely to ensure that there are no tears or perforations.

#### Turning the insulated carbon fiber cable into movement detectors:

**Figure 8:**
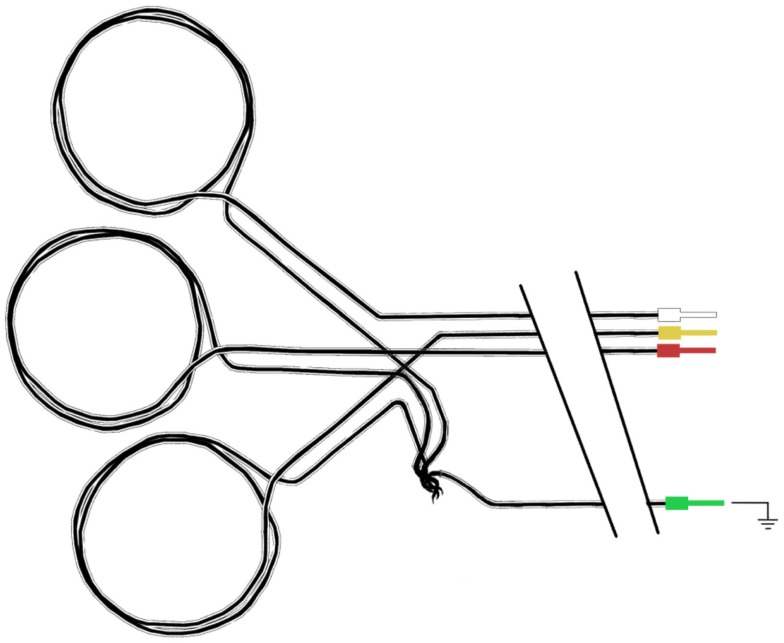
**Schematic diagram of carbon fiber movement loops**. Each loop has a diameter of approximately 5 cm, and all three loops share a common ground carbon fiber wire.

We use three movement detection loops to ensure artifact signal arising from movement in all three spatial dimensions can be captured. We have made them so that they can be individually positioned on the patient’s head in an approximately orthogonal spatial arrangement. To make the three movement sensors, four carbon fiber cables are used. Three (the sensors) have double loops introduced near the head end (with a diameter of around 5 cm) and the fourth (the ground) remains straight. After each movement sensor cable leaves the double loop, the exposed carbon fiber is twisted with the common ground fiber (Figure 8) and these are secured as best as possible and completely insulated with layers of heat-shrink tubing.

**Figure 9:**
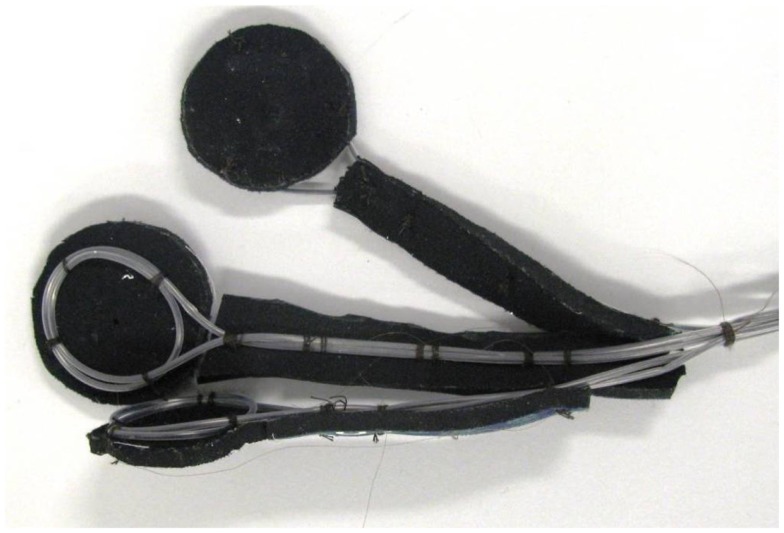
**Photograph of completed carbon fiber movement detection loops**.

Due to the large loops of carbon fiber wire used in these motion detectors, there is an increased chance of heating or current leakage compared to conventional EEG electrodes. Therefore, we take an extra precaution and place the movement loops on a bed of neoprene of at least 4 mm thickness (4) (Figure 9).

Aside from the large intentional loops insulated on the bed of neoprene, it is important that we have no other loops near the patient that may compromise safety. Therefore, we construct a full lead set and adjust the length of each individual cable to minimize slack. We also plait the cables (this is critically important for EEG electrodes to reduce the gradient artifact as it minimizes loop area and provides some cancelation of currents that would otherwise arise from the remaining small loop area; it may be less important for motion loops since they are designed to measure artifact, however, it does help keep the cable organized). It is important to ensure that there is enough length of cable on each individual motion loop before plaiting starts to accommodate the largest of head sizes. We have studied over 250 people from 4 to 60 years of age with widely different head sizes and shapes with the same motion-loop set. Our plaiting starts a short distance (15–20 cm) from the vertex of the head with our current MRI head-coil configuration; the electrodes will leave the head from the vertex and travel down the bore of the magnet away from the patient. We use cotton thread to tie the cable bundle together at each end to stop the plaiting from unwinding, and also periodically along the length of the bundle to keep it tight.

Once all the cables are plaited and bundled together, they may end up having slightly different lengths. We trim some of the longer ones at this stage so that our final connections will be tidy. We also label each cable at each end to aid troubleshooting. We do this using printed paper labels that we slip under a section of clear heat-shrink tubing (these labels will survive our normal between-subject cleaning protocol). The labeling must be completed before the addition of components at the amplifier end of the cable. Applying the heat shrink can be tricky because the PE tubing will deform if it gets too warm. It can take a little practice to get this right, so we practice on some offcuts first until we are comfortable with the process.

#### Connecting to the amplifier

At the amplifier end of each cable, we attach an RF absorber to limit RF contamination of the signal of interest. This is particularly important for EEG electrodes as the absorber will filter noise from the EEG electronics escaping the EEG shielded box, and present high impedance to the patient. For the motion loops, we ideally want the artifact measured to be similar to that contaminating the recorded EEG, so we use a similar RF absorber. Note that the amplifier in our setup is sufficiently far from the head and outside the bore of the magnet so we can use a small amount of non-magnetic metal that will not compromise the imaging.

We also need to attach the carbon fiber to some sort of input plug for the amplifier. We do this by physically crimping the carbon fiber onto a ferrule threaded over the carbon fiber and inserted into the bore of the PE tubing. In order to make this connection more secure, we also fold the carbon fiber back on itself and hold it in place with some heat-shrink tubing. These steps are explained in more detail below.

**Figure 10:**
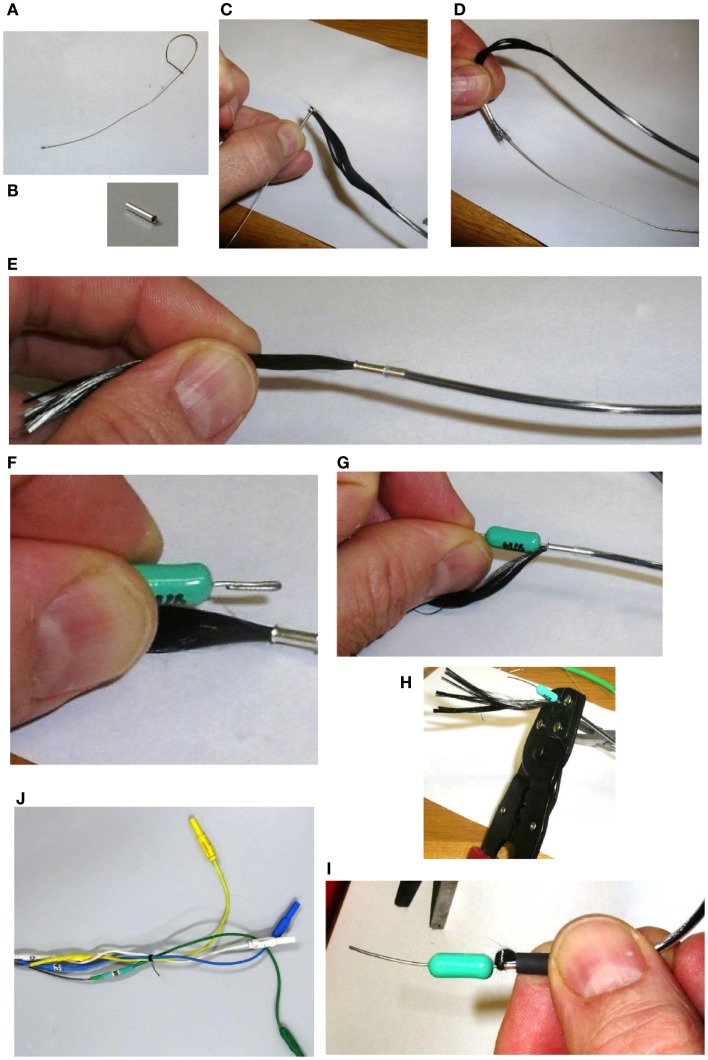
**Assembling the amplifier end of the carbon fiber cable**. First, we thread a small length of heat shrink tubing onto the carbon fiber cable. Then a small guide wire **(A)**, is fashioned into a hook and used to thread the carbon fiber through a ferrule **(B)**, by catching the very end of the carbon fiber in the hook **(C)**, and carefully pulling the wire hook through the ferrule **(D)**. We feed the carbon fiber through the ferrule until it is up against the PE tubing, and then push the ferrule into the PE tubing **(E)**. Next, we fold the wire coming out of the RF Absorber and push that into the ferrule **(F,G)**. We then use crimping pliers to firmly crimp the ferrule onto the carbon fiber/RF Absorber wire **(H)**. We trim the excess carbon fiber and thread it back through the heat shrink tubing that we put on the electrode cable earlier. We push this up to the RF Absorber **(I)**, and then apply some heat to the heat shrink tubing to hold the excess carbon fiber firmly. Finally, we solder a short color- coded wire to the other end of the RF Absorber and connect the wire to a touch-proof connector **(J)**.

First, we thread a small length of heat-shrink tubing onto the carbon fiber cable. Then, we draw the carbon fiber through the ferrule. While it might be possible to simply push the carbon fiber through the ferrule without a guide wire, we found this very difficult. Therefore, we again utilize the wire hook technique: for this purpose, we make another, smaller guide wire (Figure 10A). We thread the wire hook through the ferrule, and then catch the very end of the carbon fiber in the hook (Figures 10B,C). We then carefully pull the wire hook through the ferrule and the fiber comes with it (Figure 10D). We feed the carbon fiber through the ferrule until it is up against the PE tubing, and then push the ferrule into the PE tubing. If chosen appropriately, the ferrules fit snugly into the bore of the PE tubing (Figure 10E). Next, we fold the wire coming out of the RF absorber and push that into the ferrule (Figures 10F,G). We then use crimping pliers to firmly crimp the ferrule onto the carbon fiber/RF absorber wire (Figure 10H). Finally, we trim the excess carbon fiber and thread it back through the heat-shrink tubing that we put on the electrode cable earlier. We push this up to the RF absorber (Figure 10I) and then apply some heat to the heat-shrink tubing to hold the excess carbon fiber firmly. We make sure that there is no carbon fiber exposed outside of the heat-shrink, as this may cause the cable to short out if it touches any other conducting surface. We sometimes need to use another piece of heat-shrink to cover any loose ends.

We now have a piece of wire (the other end of the RF absorber) connected to the carbon fiber cable that we can solder things onto. We use short color coded wires, soldering one end to the RF absorber and the other end to the connector (Figure 10J). We also label the individual wires to help with troubleshooting and cover any exposed surfaces with heat-shrink tubing (remembering to slip the heat-shrink over the wire before soldering the final end of the wire on to the connector).

## Methods (Application)

We have previously described the principle of operation and validated the use of our motion loops for reduction of motion artifact when used with our in-house EEG–fMRI system (4). We take the opportunity in the present technical note to show that the system can also be successfully employed in conjunction with a commercially available fMRI-compatible EEG system: BrainAmp MR from Brain Products GmbH.

### Subjects

A healthy male subject aged 25 years and a female epilepsy patient aged 12 years, were studied. The patient had experienced seizure onset at the age of 3 years with electrographic diagnosis of continuous spikes and waves during sleep (CSWS) at age of 6 years. Ethical approval for this study was obtained from the Austin Hospital Human Research Ethics Committee. Written informed consent was obtained from the healthy subject and the father of the patient.

### Data acquisition

Functional MRI of the healthy subject was acquired with a Siemens MAGNETOM TRIO MRI scanner (Siemens Medical Solutions, Erlangen, Germany) equipped with a Siemens Tx/Rx CP Head Coil. A gradient-echo echo-planar imaging (EPI) sequence was utilized with TR = 3 s; TE = 30 ms; flip angle = 85°; FOV = 216 mm × 216 mm; 72 × 72 matrix; voxel size 3 mm × 3 mm × 3 mm; 44 contiguous slices 3 mm thick, providing whole-brain coverage. Two hundred T2*-weighted whole-brain volumes were acquired in a 10 min scanning session.

Functional MRI of the patient was acquired with a Siemens MAGNETOM Skyra MRI scanner (Siemens Medical Solutions, Erlangen, Germany) with an otherwise similar setup to that described above. Six hundred T2*-weighted whole-brain volumes were acquired in a 30 min scanning session.

**Figure 11:**
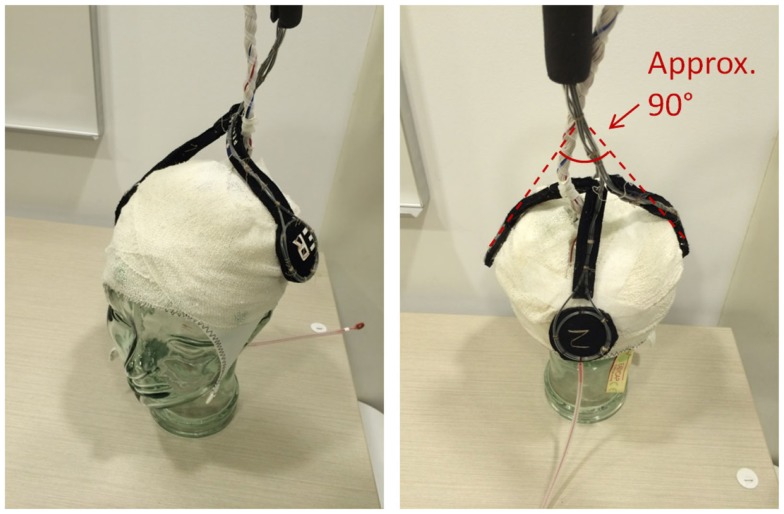
**Precise positioning of the motion loops is not critical; however, they should be placed on the head in an approximately orthogonal orientation, so they capture the effect of motion in any direction (i.e. no loop should be parallel to another)**.

**Figure 12:**
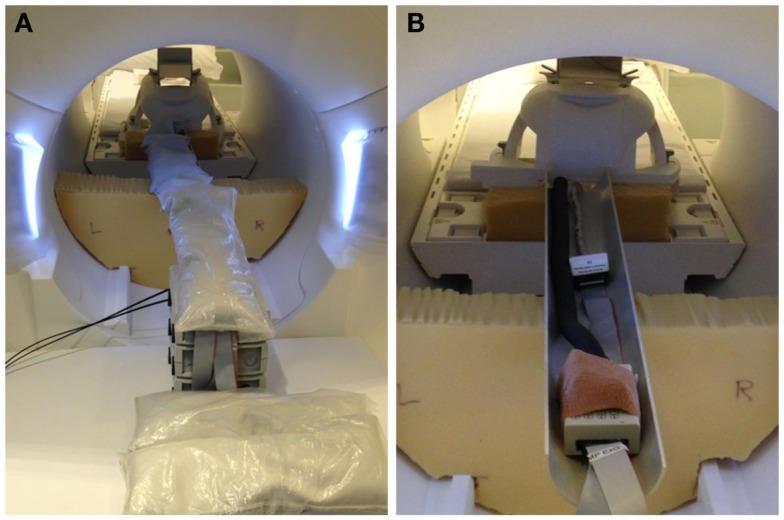
**Photographs of inside-MRI-scanner EEG equipment layout similar to that utilized in the present experiment**. To obtain a clear view, these photographs were taken in our mock-scanner; in our real scanner, we use a head coil that permits entry of the cables directly from the rear. **(A)** The EEG amplifier and peripheral signal amplifier are both placed outside the scanner bore and the cable connecting the EEG cap to the amplifiers fixed in place in the center of the bore using plastic piping covered with sandbags, suspended on pieces of thick foam padding. **(B)** View of the cables with the sandbags removed.

EEG for both subjects was acquired using a Brain Products MR-compatible EEG system configured for 32-channel operation (BrainCap MR from EASYCAP GmbH). The cap is fitted with 32 electrodes (including the reference) with sintered Ag/AgCl sensors. Electrodes were arranged according to the international 10–20 system. Electrocardiogram was recorded using an electrode placed on the subject’s back. Head movement detection loops (permanently attached to a bed of 4 mm thickness neoprene as shown in Figure 9) were placed on top of the EEG cap such that loop orientations were approximately mutually orthogonal. As a convenience measure, prior to placing the loops, we wrapped the electrode cap with a bandage to avoid getting surplus electrode gel on the motion loops (Figure 11). The loops were then affixed in place with further bandages. The EEG amplifier (BrainAmp MR, Brain Products GmbH) and peripheral signal amplifier used for the motion loops (BrainAmp ExG MR, Brain Products GmbH) were placed outside the scanner bore. The cables connecting the EEG cap and motion loops to the amplifiers were run down the center of the scanner bore, fixed in place using plastic piping and sandbags (Figure 12). The amplifiers were connected via fiber optic cabling to a computer outside the scanner room. The EEG clock was synchronized with the MRI scanner’s clock using Brain Products’ SyncBox. EEG was acquired using BrainVision Recorder using a sampling rate of 5000 Hz.

### Healthy subject paradigm

EEG was first recorded for 10 min outside the scanner. The subject was verbally instructed to open and close eyes for alternating periods of 30 s. The subject was then moved to the MRI scanner where they were verbally instructed to open and close eyes for alternating periods of 30 s for the first 5 min of the fMRI scanning. For the next 5 min, the subject was instructed to keep their eyes closed. This 10 min paradigm was then repeated in a second study within the same scanning session, with the subject additionally instructed to occasionally move their head at random times of their choice throughout the scan.

### Epilepsy patient paradigm

EEG was first recorded for 10 min outside the scanner to capture the morphology and distribution of epileptic discharges. The patient was instructed to keep their eyes closed for the duration of the recording. The patient was then moved to the MRI scanner where they were instructed to close their eyes during scanning, and encouraged to fall asleep.

### Offline EEG analysis

Offline analysis of the EEG data was performed using BrainVision Analyzer 2.0 software as follows:

1. Removal of MR gradient artifact from the EEG and motion-loop signals using a sliding average artifact template subtraction method. Twenty-one volume intervals (each corresponding to one TR = 3 s) were used to compute each average.
2. EEG and motion-loop signals downsampled to 250 Hz.
3. EEG and motion-loop signals low- and high-pass filtered at 70 and 0.5 Hz, respectively (Butterworth zero-phase filter, 48 dB/octave).
4. Motion/cardioballistic artifact (CBA) correction was then performed using two different methods, each in separate analysis streams that could be subsequently compared. The first method is a conventional method that does not utilise the motion loops. The second method utilises the motion loop signals. The methods are described below.

Method 1: cardioballistic artifact removal using BrainVision Analyzer algorithm. CBA removal was performed by identifying *R*-peak markers of each QRS complex from the ECG channel (*R*-peak search parameters: 60–100 pulses/min; average pulse length 800 ± 200 ms), and then performing a sliding average artifact template subtraction (25), with each subtraction template consisting of 21 *R*-peak intervals. The delay time (i.e., the time between the *R*-peak of the ECG and the CBA peak in the EEG trace) was computed across the whole EEG recording and used to center the artifact correction template to improve correction of each CBA episode (average delay time was 0.408 s for the healthy control, and 0.100 s for the patient).
Method 2: cardioballistic/motion artifact removal utilizing signals derived from the three motion loops. An estimate of the artifact contained in each recorded EEG channel was derived from the motion-loop signals and then subtracted from the EEG (4). In the present implementation, this was achieved using a multi-channel least squares algorithm implemented as an external custom MATLAB procedure called from BrainVision Analyzer (see Appendix).

We then took additional steps to check for and, if necessary, mitigate the effect of motion deleteriously affecting the average-gradient-artifact correction procedure. We first checked for the presence of large EEG signal likely due to motion so extreme that the motion-loop procedure was unable to remove it from the EEG. Specifically, we used the automated “raw data inspection” procedure available in BrainVision Analyzer software, applied to the motion-corrected EEG channels, to automatically search and mark periods of large EEG signal change as bad. Three criteria were used as follows: (i) “Check Gradient” was used to detect EEG signal amplitude steps >50 μV/ms and these were marked as bad commencing 200 ms before and concluding 200 ms after each instance; (ii) “check min/max amplitude” was used to mark as bad time intervals of 200 ms in which the maximum–minimum amplitude exceeded a threshold of 200 μV; (iii) “check minimum and maximum allowed amplitude” was used to mark as bad any instance of values lower than −200 μV or higher than +200 μV, with the excluded window commencing 200 ms prior and extending 200 ms after the deleterious event. If any epochs of large amplitude/change were identified, the gradient artifact subtraction was re-done as follows: the markers were upsampled to 5 kHz to match the original EEG acquisition. The gradient artifact correction steps 1–3 above were then re-done on the original uncorrected EEG and motion-loop signals, informing the procedure not to use the marked-as-bad epochs when generating average gradient correction templates. Finally, the (possibly improved) motion-loop signals were then inspected using the “raw data inspection” routine to detect significant motion (using a tighter constraint for “Check minimum and maximum allowed amplitude,” marking as bad any instance of values lower than −100 μV or higher than +100 μV). If any was found in epochs not already marked as bad, the gradient artifact correction steps 1–3 above were re-done once more, this time excluding the expanded set of bad epochs. An estimate of the artifact contained in the newly processed EEG signals was then derived from the newly processed motion-loop signals and the artifact was subtracted as before.
Note that the voltage settings that we used in the “raw data inspection” step were chosen heuristically. Appropriate settings are likely to vary between systems. For example, the voltages returned by the motion loops will depend upon the area and number of turns of the constructed loops as well as the magnetic field strength of the MRI.

## Results

The measured resistance of a single electrode included with our BrainAmps system was 10 kΩ. Thus, a circuit consisting of two electrodes would be at least 20 kΩ. Allowing for scalp impedance (typically 8–10 kΩ as measured by the BrainAmps equipment at 15 Hz) and a comfortable margin, we selected a 32 kΩ resistor to use in series with each of our motion loops.

**Figure 13:**
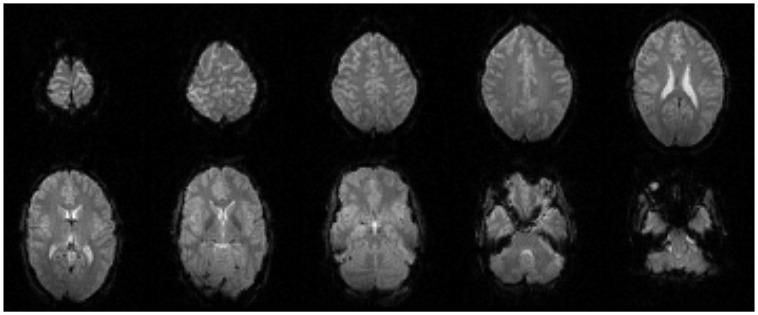
**Every fourth slice of an EPI volume acquired from the healthy subject while simultaneous EEG recording was in progress**.

**Figure 14:**
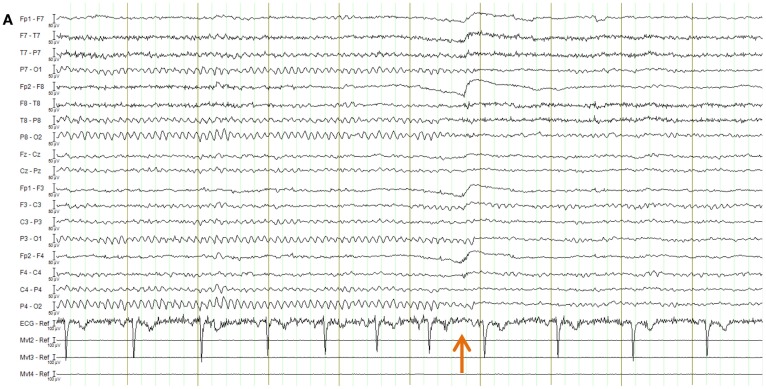

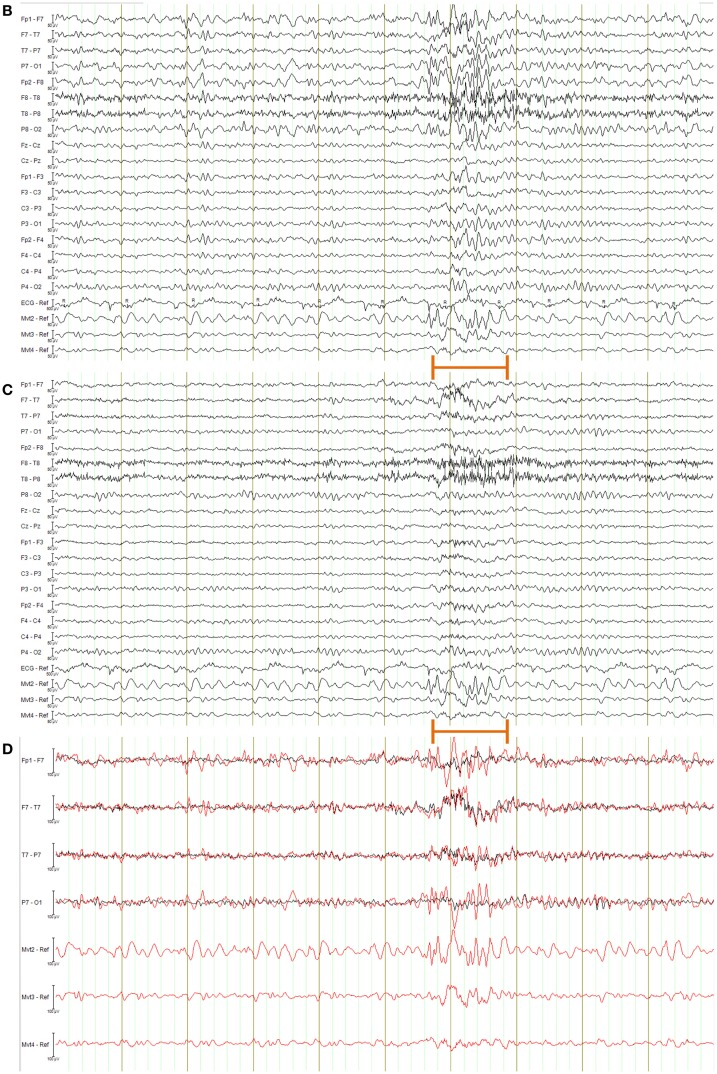
**EEG of the healthy subject shown in longitudinal bipolar (“double banana”) montage**. In every figure **(A–D)**, the lower three traces are motion-loop signals. **(A)** Ten second segment of EEG recorded outside the MRI scanner selected to show eyes closed then, following the time point indicated by the arrow, eyes open. Typical prominent alpha activity in the absence of MRI artifact is clearly evident while the subject’s eyes are closed; a brief blink artifact is then evident as the subject opens their eyes at the time indicated by the arrow, followed by reduced alpha activity during the eyes open condition. Any motion of the subject is not detectable in the motion loops because they are not in a magnetic field. **(B–D)** a 10 s segment of eyes-closed EEG recorded inside the scanner during fMRI acquisition, selected to include an obvious large movement event at the time indicated by the orange bar: **(B)** result when the EEG is corrected using conventional average-pulse-artifact subtraction; **(C)** result when motion-loop artifact removal is applied; **(D)** expanded overlay of four selected EEG channels more clearly demonstrating the superiority of the motion-loop artifact removal technique (black trace) compared to conventional pulse-artifact subtraction (red trace).

**Figure 15:**
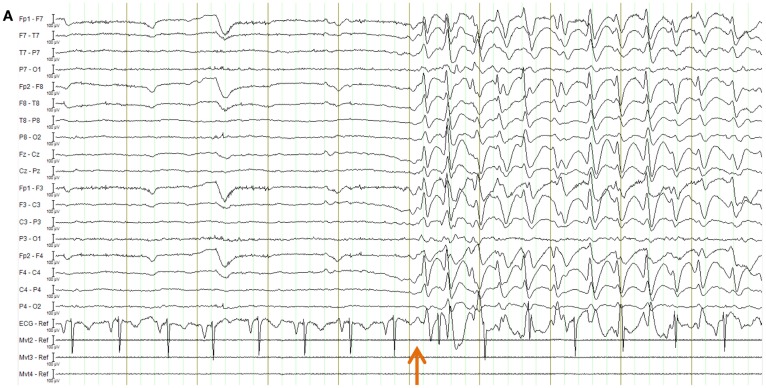

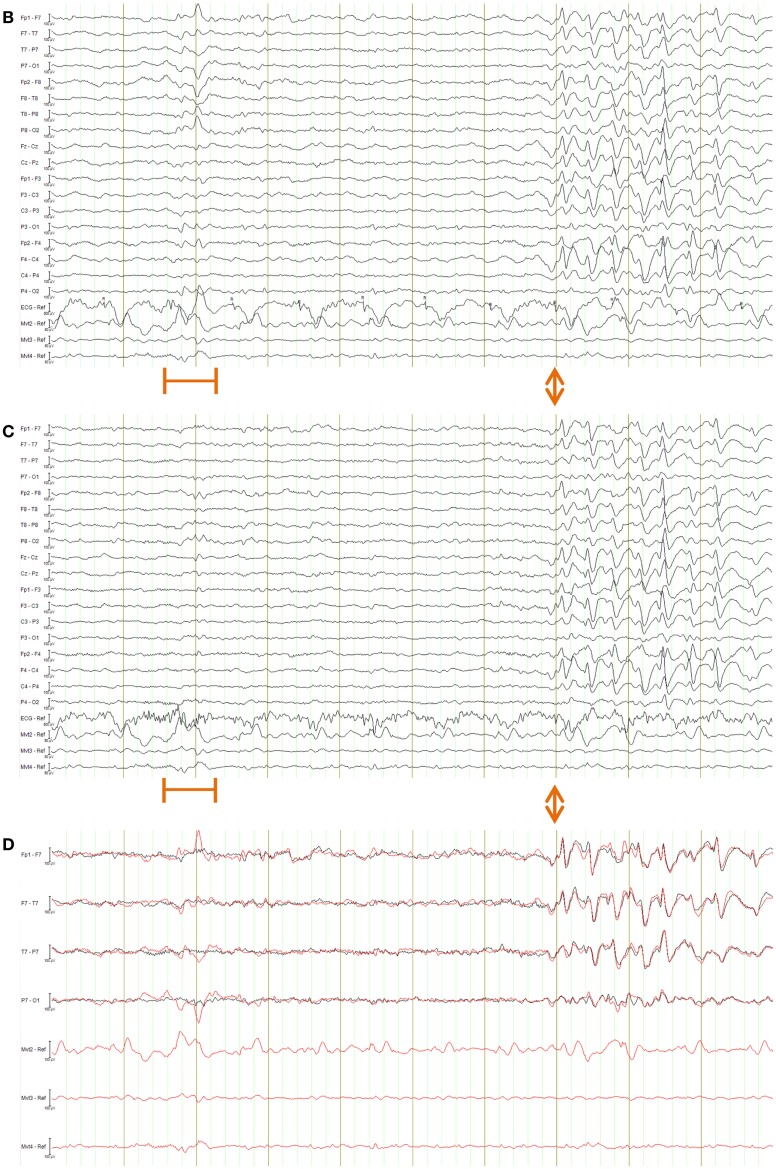
**EEG of the epilepsy patient shown in longitudinal bipolar (“double banana”) montage**. In every figure **(A–D)**, the lower three traces are motion-loop signals. **(A)** Ten second segment of EEG recorded outside the MRI scanner selected to show a typical epileptiform discharge of this patient (commencing at the time indicated by the arrow) in the absence of MRI artifact. Any motion of the subject is not detectable in the motion loops because they are not in a magnetic field. **(B–D)** a 10 s segment of EEG recorded inside the scanner during fMRI acquisition, selected to include both a large movement event (at the time indicated by the orange bar) and a typical epileptiform discharge of this patient (commencing at the time indicated by the double arrows): **(B)** result when the EEG is corrected using conventional average-pulse-artifact subtraction; **(C)** result when motion-loop artifact removal is applied to the EEG – notice the large motion artifact early in the record is removed, as is cardioballistic artifact throughout the study, while epileptiform activity evident later is retained (the BrainAmps system also effectively removed the cardioballistic artifact in this example, however, it failed to remove the non-periodic motion event); **(D)** expanded overlay of four selected EEG channels more clearly demonstrating the superiority of the motion-loop artifact removal technique (black trace) compared to conventional pulse-artifact subtraction (red trace).

**Figure 16:**
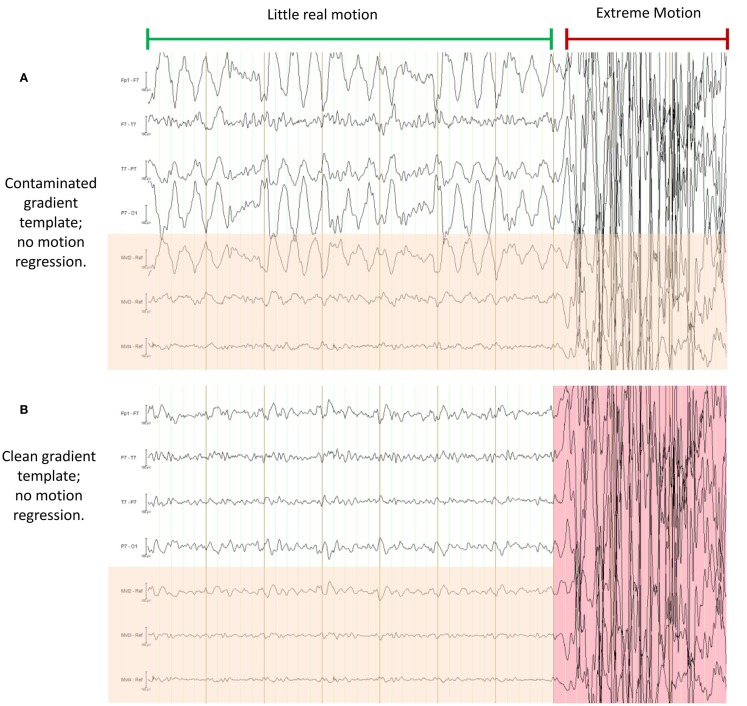

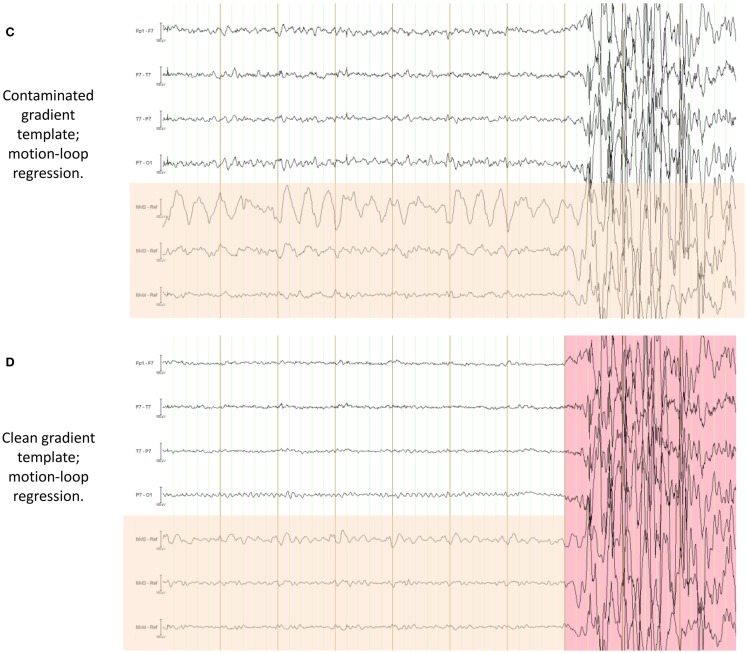
**This figure demonstrates a particularly severe example of deliberate motion contamination in the healthy control in which gross motion has occurred to such an extent that even the fraction 1/21 is substantial and has resulted in propagated artifact due to contamination of the average-gradient-artifact template (the template being an average of 21 epochs of length TR)**. In each of **(A)** through **(D)** the same 10 s period is shown with different processing applied; in each figure, the upper four traces are selected EEG traces, the lower three traces (with orange shading) are the corresponding motion-loop signals. The extreme motion event occurred during the time marked by the red indicator line. **(A)** is the conventional result without motion-loop subtraction. In this result, it appears as if there is also less intense yet still substantial motion contaminating the entire time shown prior to the extreme motion event (i.e., during the epoch indicated by the long green indicator line). In **(B)**, the gradient artifact correction has been re-run, excluding the use of the severe motion event from the gradient artifact correction template (i.e., the epoch highlighted in pink was excluded). This has substantially improved the remainder of the displayed EEG. Notice also that the motion-loop signals leading up to the extreme motion event no longer exhibit large motion signal, confirming the large apparent motion in the green epoch seen in **(A)** was actually due to a contaminated gradient correction template. **(C)** is the result of the first iteration of the motion-loop correction procedure, i.e., the same contaminated EEG shown in **(A)** has simply been subjected to motion-loop subtraction: the extreme motion event was too large to be completely removed (suggesting substantial non-linear effects); however, the smaller propagated artifact has been virtually eliminated from the EEG signal, and cardioballistic artifact is also reduced, so the EEG during the green labeled epoch is noticeably cleaner in **(C)** than in **(B)**, even though the gradient artifact correction template remains contaminated in **(C)**. Finally in **(D)**, the iterative process has been applied: the bad epoch was automatically identified from the result of the first iteration, then a second gradient artifact correction was applied to avoid motion contamination of the gradient artifact correction template, and finally motion-loop regression was applied to this improved data. This provides the cleanest EEG signal of all.

EPI image quality with the EEG leads and motion loops in place was acceptable (Figure 13). There were no adverse effects related to the use of the motion loops during this or any other study at our site. Segments of EEG demonstrating the performance of each motion removal method are displayed for the healthy subject in Figure 14 and for the epilepsy patient in Figure 15. A particularly extreme motion event example from the second study undertaken by the healthy control is also shown in Figure 16 to specifically demonstrate the ability of motion-loop correction to reduce propagated artifact related to motion contamination of the average-gradient-artifact correction template.

## Discussion

We have described the methods that we use to construct and utilize carbon fiber motion loops with a commercially available EEG–fMRI system. We observe that the quality of the EEG corrected by motion-loop signals is often superior to that of conventional software-only correction methods, especially in the presence of non-periodic motion. When applied in the study of epilepsy, the motion loops also confer the advantage of displaying a direct measure of artifact to the EEG reader, providing information that can increase confidence in EEG mark-up.

### Mitigating problems with gradient artifact correction

We have previously described the use of motion loops in conjunction with an EEG–fMRI system that we built in-house. In that EEG–fMRI system, gradient artifact was largely avoided during acquisition of the EEG, so average-gradient-artifact post-processing was not required. In current commercially available systems, a different approach is taken to gradient signals: the EEG including gradient artifact is measured in its entirety. A gradient-artifact-removal post-acquisition processing step is then performed – typically a gradient artifact waveform template of temporal length TR (the MRI repetition time) is determined by averaging the EEG over a number of successive time windows each of length TR. This can provide a good estimate of the gradient artifact, while the physiological signals of interest tend to average close to zero in the template. However, subject motion can contaminate the estimate of the average gradient artifact. This can then degrade the corrected EEG for the entire time period in which the affected average gradient template is used. A particularly severe example of this is shown in Figure 16A – gross motion has occurred to the extent that even the fraction 1/21 of the resultant artifact is substantial and this fraction has been propagated to surrounding epochs during the average template subtraction procedure (the template being an average of 21 epochs of length TR). This type of artifact also substantially affects gradient correction of the motion-loop signals, so it is difficult to be sure whether or not the motion-loop signals and EEG contain propagated or real motion or both. In the case of Figure 16, exclusion of the extreme epoch worked well to avoid large contamination of the average-gradient-artifact correction, as shown in Figure 16B. There are several methods available to help identify and remove such extreme epochs [for a comparison of several gradient-artifact-removal algorithms, see Ref. (26)]. However, the intent of this motion example is to illustrate that the motion loops alone can mitigate failures in gradient artifact removal, and assist with identification of real motion events. Figure 16C demonstrates that subtraction of the fitted motion-loop signals has alone almost completely removed the artifact that was propagated during gradient artifact pre-processing, even in this extreme example. The noise reduction benefits of the motion-loop subtraction method therefore include reduction of any directly measured motion artifact and reduction of artifact resulting from imperfect gradient artifact removal. In practice, we recommend an iterative procedure as this process yields the cleanest EEG (e.g., Figure 16D). The second iteration avoids average gradient template contamination by extreme events identified after the first iteration. This iterative process is more straightforward when motion loops are used because there can be a clearer distinction in the EEG between potential non-motion epochs and extreme real motion epochs (compare, for example, Figures 16A,C). The final iteration avoids average gradient template contamination by more subtle motion events that can now be identified from the improved motion-loop signals of the second iteration, because the motion loops are no longer substantially contaminated by propagated artifact from the more extreme events. The more subtle events may not have been detected in the EEG at the first iteration because they are more effectively removed by the motion-loop subtraction. Of course, the average gradient template subtraction procedure only needs to be re-run at each of these stages if additional bad epochs are actually detected.

Due to the high amplitude, high frequency, and highly consistent periodic nature of the gradient artifact, the procedure described above is substantially more effective than attempting to correct the entire gradient artifact by direct regression of the three motion-loop measurements. However, if residual gradient artifact is present in the EEG after this procedure (even if not as obvious as the extreme motion example presented in Figure 16), it will also be present in the motion-loop signals and so will be further attenuated by the subsequent motion-loop artifact regression procedure.

### Limitations and potential to detect rapid motion that may also affect fMRI

We have previously shown that if motion artifact is too extreme, the motion-loop correction procedure is unable to effectively remove the contamination (although the motion loops do at least alert the user to the presence of motion in those circumstances) (4). Figure 16 provides another demonstration of this limitation: while the propagated artifact could be removed, in this example, the epoch in which the actual motion event occurred could not be adequately corrected, suggesting substantial non-linear effects. We wondered how much motion had occurred during this epoch. It is not possible to determine the absolute amount of motion from our uncalibrated motion-loop signals. The magnitude of the artifact depends upon the rate of change of magnetic flux through the loop formed by the conductor, which in turn depends upon both the rate of movement and its direction with respect to the magnetic field. However, we can obtain a crude estimate of the actual motion from the fMRI acquisition. We determined the within-brain center of intensity change of the fMRI for the extreme motion epoch of Figure 16 using iBrain software (27)^3^: the change in 2D center-of-mass (intensity) of each slice in the affected volume, compared to the same slice in the previous volume, ranged from 0.14 to 2.1 mm, while the change in 3D center-of-mass between volumes was 0.95 mm. We also estimated 3D shift and rotations using the rigid-body realignment procedure in SPM software^4^. The volume-to-volume change in displacement estimated this way was 0.76 mm accompanied by a change in pitch rotation of 0.68°. The peak motion detected in 2D slices in this example is thus considerably larger than volumetric parameters would suggest (i.e., motion was sufficiently rapid to affect slices within the volume differently, and this effect was partially averaged out in the rigid-body motion estimates across the entire volume). Given the relatively high temporal sampling rate of the EEG compared to the volume or slice acquisition time of fMRI, the motion-loop signals may have additional potential to be used to identify fMRI volumes that may be affected by rapid subject motion. For example, the inability of the motion loops to adequately remove motion artifact could be used as an indicator of the presence of motion severe enough to result in non-linear EEG artifact, and therefore, also likely to have a deleterious effect on the fMRI acquisition at that particular time. We recommend further work be undertaken to explore this potential.

### Safety

We remind the reader again that wires in an MRI scanner can be very dangerous if the proper precautions are not taken. For simultaneous EEG–fMRI experiments conducted with our leads, we have always used MRI head-coils that enable the EEG wires to leave at the vertex of the head and travel directly away from the patient to the back of the scanner bore, rather than enclosed coils that would require leads to run back alongside the patient. It is likely that further insulating safety measures would be required to render these cables safe in the latter configuration.

## Conflict of Interest Statement

The authors declare that the research was conducted in the absence of any commercial or financial relationships that could be construed as a potential conflict of interest.

## Footnotes

^1^http://www.microtube.com.au/

^2^http://au.element14.com/

^3^http://www.brain.org.au/software

^4^http://www.fil.ion.ucl.ac.uk/spm

## Appendix

External MATLAB routine used to remove motion-loop artifact signal from each EEG channel:

% START MATLAB CODE
  motion_channels = [34:36];
 
  window_length = 2;
 
  filter_channels = [1:numel(Properties.Channels)];
  filter_channels(motion_channels) = [];
 
  width = window_length*(1000000/Properties.SamplingInterval);
  window = [0:width-1];
  step = width;
 
  for e = 1:step:(Properties.DatasetLength-width)
 
    template = EEGData(e+window,motion_channels)’;
    template = template - mean(template,2)*ones(1,width);
 
    ptemplate = pinv(template’);
 
    for c = 1:numel(filter_channels)
 
     artifact = EEGData(e+window,c)’;
     beta = ptemplate*(artifact - mean(artifact))’;
     filtered = artifact - beta’*template;
 
     EEGData(e+window,filter_channels(c)) = filtered’;
 
  end
end
% END MATLAB CODE
